# Inequality of weight status in urban Cuba: 2001–2010

**DOI:** 10.1186/s12963-021-00251-6

**Published:** 2021-05-04

**Authors:** Peng Nie, Lanlin Ding, Alfonso Sousa-Poza, Alina Alfonso Leon, Hong Xue, Peng Jia, Liang Wang, Youfa Wang

**Affiliations:** 1 School of Economics and Finance, Xi’an Jiaotong University, Xi’an, 710061 Shaanxi China; 2 Institute for Health Care & Public Management, University of Hohenheim, Stuttgart, Germany; 3 Global Health Institute, School of Public Health, Xi’an Jiaotong University Health Science Center, Xi’an, 710061 Shaanxi China; 4 Institute of Labor Economics (IZA), Bonn, Germany; 5 Centre for Demographic Studies (CEDRM), University of Havana, Havana, Cuba; 6 Department of Health Administration and Policy, College of Health and Human Services, George Mason University, Fairfax, VA 22030 USA; 7 Department of Land Surveying and Geo-Informatics, The Hong Kong Polytechnic University, Hong Kong, China; 8 International Institute of Spatial Lifecourse Epidemiology (ISLE), Hong Kong, China; 9 Department of Public Health, Robbins College of Health and Human Sciences, Baylor University, Waco, Texas, USA

**Keywords:** Body mass index, Waist circumference, Obesity, Inequality, Decomposition, Urban Cuba

## Abstract

**Background:**

Although understanding changes in the body weight distribution and trends in obesity inequality plays a key role in assessing the causes and persistence of obesity, limited research on this topic is available for Cuba. This study thus analyzed changes in body mass index (BMI) and waist circumference (WC) distributions and obesity inequality over a 9-year period among urban Cuban adults.

**Methods:**

Kolmogorov-Smirnov tests were first applied to the data from the 2001 and 2010 National Survey on Risk Factors and Chronic Diseases to identify a rightward shift in both the BMI and WC distributions over the 2001–2010 period. A Shapley technique decomposed the increase in obesity prevalence into a mean-growth effect and a (re)distributional component. A univariate assessment of obesity inequality was then derived by calculating both the Gini and generalized entropy (GE) measures. Lastly, a GE-based decomposition partitioned overall obesity inequality into within-group and between-group values.

**Results:**

Despite some relatively pronounced left-skewing, both the BMI and WC distributions exhibited a clear rightward shift to which the increases in general and central obesity can be mostly attributed. According to the Gini coefficients, both general and central obesity inequality increased over the 2001–2010 period, from 0.105 [95% confidence interval (CI) = 0.103–0.106] to 0.110 [95% CI = 0.107–0.112] and from 0.083 [95% CI = 0.082–0.084] to 0.085 [95% CI = 0.084–0.087], respectively. The GE-based decomposition further revealed that both types of inequality were accounted for primarily by within-group inequality (93.3%/89.6% and 87.5%/84.8% in 2001/2010 for general/central obesity, respectively).

**Conclusions:**

Obesity inequality in urban Cuba worsened over the 2001–2010 time period, with within-group inequality in overall obesity dominant over between-group inequality. In general, the results also imply that the rise in obesity inequality is immune to health care system characteristics.

**Supplementary Information:**

The online version contains supplementary material available at 10.1186/s12963-021-00251-6.

## Background

The current obesity epidemic, with over 1.9 billion overweight and 650 million obese individuals worldwide [1, 2], costs approximately $2 trillion annually or 2.8% of the world’s gross domestic product (GDP) [3]. Its prevalence is also rising, especially in North America [4], Europe [5], and Asia [6, 7], with rates quadrupling among males (3 to 12%) and more than doubling among females (7 to 16%) [8]. Most of this increase in obesity prevalence is attributable to either the entire population growing heavier (i.e., a rightward shift in the bodyweight distribution) or more rapid weight gain in one subpopulation (i.e., an increase in distributional left-skewness and thus rising obesity inequality). Although research for England and Canada has identified a polarization over time toward the right-end of the body mass index (BMI) distribution [9], studies for United States (US) have attributed the early phase of the obesity epidemic mostly to increasing skewness, but recent rises in obesity rates to a population-wide increase [10]. Recent work for China, in contrast, documented a clear rightward distributional shift combined with a leftward skewing among adults aged 20+ over the 1991–2011 period [11].

Such obesity inequality, being an important indicator of well-being, plays a pivotal role in assessing obesity’s negative social effects (e.g., discrimination and harassment), as well as adult obesity persistence [11]. Unfortunately, such inequality has worsened over time, increasing dramatically in both China and the US due primarily to rising within-group inequality. Only a limited number of studies, however, have documented the spectacular increases in Cuba, which has witnessed a sharp rise in general overweight and obesity (from 33.5% in 1995 to 52.9% in 2010) [12] accompanied by moderate growth in central obesity (from 40.0% in 2001 to 48.0% in 2010) [13]. Without effective interventions, this prevalence of general overweight and obesity is projected to increase from about 58% (67%) in 2010 to a staggering 94% (89%) in 2050 for males (females), the highest among all Latin American countries [14].

Cuba offers a particularly interesting case for studying obesity inequality because rather than experiencing major economic fluctuations, Cuba had been in constant recovery since the 1991–1995 economic crisis (referred to as the “special period”) [15, 16], with GNI per capita almost tripling between 2001 and 2010 [17]. In particular, the “special period” resulted in an average weight loss of 4–5 kg across the adult population [12, 15, 16], highlighting the important impact of macroeconomic conditions on obesity [13]. In addition, its egalitarian health care system’s provision of full access to high-capacity, good-quality primary care [18] tends to eliminate regionally heterogeneous health outcomes and ensure more equal distribution than in other countries. Yet, this latter may itself generate an erroneous assumption that obesity inequality is not a major problem in Cuba, leading to the current dearth of studies that use nationwide data to examine body weight distribution and obesity inequality over time.

To address this gap, this present study used a nationally representative survey dataset to provide evidence on the patterns and temporal changes in bodyweight distribution and obesity inequality in Cuba between 2001 and 2010. Achieving this goal involved four primary tasks: (1) analyzing the changes in both BMI and waist circumference (WC) distributions among urban Cuban adults (≥ 18 years) over 2001–2010; (2) decomposing the total change in obesity prevalence into a mean-growth and a redistribution component; (3) deriving a univariate assessment of obesity inequality based on conventional inequality measures (i.e., Gini coefficient and generalized entropy); and (4) partitioning overall obesity inequality into within-group and between-group inequality to determine whether disproportionate obesity increases are a population-wide phenomenon or the result of changing demographic composition.

## Methods and materials

### Data and study sample

The data were drawn from the National Survey on Risk Factors and Chronic Diseases (NSRFCD) in Cuba, administered collaboratively by the National Institute of Hygiene, Epidemiology and Microbiology, the National Statistics Bureau, and the Nutrition and Food Hygiene Institute [19]. This nationally representative survey, which used a stratified multistage cluster sampling design [19], was administered in all urban areas in 1995 (NSRFCD I) and 2001 (NSRFCD II) and in both urban and rural areas in 2010 (NSRFCD III). The analytic sample used for this current study was restricted to adults aged 18 and older for whom detailed demographic, socioeconomic, and anthropometric information (including weight, height, and WC) was available for two waves (NSRFCD II and III). To improve comparability with NSRFCD II data, however, the NSRFCD III data were restricted to urban areas. The final pooled sample comprised 25,195 BMI observations (20,118 and 5077 in the NSRFCD II and III, respectively) and 25,496 WC observations (20,365 and 5131 in the NSRFCD II and III, respectively), with the NSRFCD III sample being relatively smaller (*n* = 7,915) because of financial constraints. We employed data from NSRFCD II and NSRFCD III as our analytic sample for two main reasons: First, in the NSRFCD I, we found a large number of missing and implausible values of individual weight and height, which would result in biases when calculating BMI. Second, WC is only available in the 2001 NSRFCD II and 2010 NSRFCD III. We used both BMI and WC as two bodyweight measures, thereby facilitating us to compare inequalities in general obesity and central obesity during the same period.

### Outcome variables

Because BMI (in kg/m^2^) is a common proxy of body weight status, general obesity was defined based on the World Health Organization (WHO) criterion of BMI ≥ 30 kg/m^2^ [12, 13, 20]. BMI, however, gives no indication of fat distribution [21, 22], so WC, defined according to the International Diabetes Federation (IDF) criteria of WC ≥ 90 cm (80 cm) for males (females), served as the proxy for central obesity [18]. We used both BMI and WC as two bodyweight measures mainly because BMI does not capture the distribution of body fat, which can give rise to misleading results. WC is a more accurate measure of the distribution of body fat and has been shown to be more strongly associated with morbidity and mortality [23, 24].

### Sociodemographic variables

To detect subpopulation heterogeneity in obesity inequality, the analysis employed several sociodemographic characteristics, including gender (male, female), age group (18–39, 40–59, 60+ years), race (White, Mulatto, Black), marital status (single, married/living together, widowed/separated/divorced), and educational level (low: illiterate/primary school; medium: secondary school/qualified worker/technical school; high: university and above).

### Statistical analyses

Once the 2001–2010 distributional changes in BMI and WC were expressed as kernel densities (i.e., nonparametric smoothed graphs independent of bin width) [25], Kolmogorov-Smirnov tests (Stata *ksmirnov* procedure) [26] determined whether entire distributions of the body weight measures had shifted rightward over the study period. A Shapley decomposition of the total change in obesity prevalence into a mean-growth and a redistribution component (see Additional file 1) then allowed assessment of how much obesity increase was driven by a horizontally shifting body weight distribution (i.e., an increase in mean BMI or WC) and how much by a changing distribution pattern (e.g., increased skewness toward the upper tail of the BMI or WC distribution) [10]. To make the data from NSRFCD II and NSRFCD III comparable, the analytic samples were weighted to ensure nationally representative estimates [20].

Tracking of the cardinal changes in obesity inequality was enabled by the introduction of Gini and generalized entropy (GE) measures (see Additional file 1). Because the GE(*ω*) indices (whose scaling parameter *ω* represents the weight assigned distances between individual BMI at different parts of the BMI/WC distribution) tended to vary in their sensitivities to differences in different distributional areas [27, 28], in subsequent robustness checks, *ω* was set to 0 and 2, enabling comparison with outcomes for the US population [10]. Note that the health inequality toolbox like the Concentration Index considers the joint distribution of health and socioeconomic rank and such bivariate rank dependent indices should be thought of as two-dimensional indices that consider the covariate between rank and health, which is beyond the scope of this study.

Lastly, a GE-based decomposition that split the GE index into within-group and between-group inequality (see Additional file 1) assessed whether changes in overall obesity inequality were being driven by changing subpopulation characteristics or a population-wide shift in bodyweight distribution [11]. Subpopulation heterogeneities were identified using a decomposition analysis of the Theil index (GE(1)) by age, gender, race, marital status, and education, as well as combinations of these categories. All the above analyses were conducted using Stata 14 [29].

## Results

### Study population characteristics

Over the 2001–2010 period, BMI increased by approximately 0.6 kg/m^2^, WC by 2.4 cm, general obesity by nearly 4%, and central obesity by around 8% (Table 1).

**Table 1:** Study population characteristics

Variables	2001			2010			Mean differences
	Mean/%	SD	*n*	Mean/%	SD	*n*	
Bodyweight weight							
BMI (kg/m^2^)	24.795	4.919	20118	25.354	5.046	5077	0.559^***^
WC (cm)	82.068	12.236	20365	84.491	12.778	5131	2.423^***^
General obesity	0.125	0.331	20118	0.163	0.370	5077	0.038^***^
Abdominal obesity	0.405	0.491	20365	0.484	0.500	5131	0.079^***^
Gender							
Male	0.470	0.500	21510	0.456	0.498	5685	− 0.015^**^
Age group							
18–39	0.473	0.500	21510	0.353	0.478	5685	− 0.120^***^
40–59	0.325	0.468	21510	0.385	0.487	5685	0.060^***^
60+	0.202	0.401	21510	0.262	0.440	5685	0.061^***^
Race							
White	0.683	0.465	21510	0.662	0.473	5685	− 0.022^***^
Mulatto	0.208	0.406	21510	0.229	0.420	5685	0.021^***^
Black	0.109	0.311	21510	0.110	0.312	5685	0.001
Marital status							
Single	0.181	0.385	21510	0.235	0.424	5685	0.054^***^
Married/living together	0.642	0.479	21510	0.595	0.491	5685	− 0.047^***^
Widowed/separated/divorced	0.176	0.381	21510	0.170	0.376	5685	− 0.006
Education							
Low	0.276	0.447	21510	0.178	0.382	5685	− 0.098^***^
Middle	0.428	0.495	21510	0.432	0.495	5685	0.004
High	0.297	0.457	21510	0.391	0.488	5685	0.094^***^

*BMI* body mass index, *WC* waist circumference. Education level: low (illiterate/primary school), medium (secondary school/qualified worker/technical school), and high (university). ^**^ and ^***^ indicate *p* < 0.05 and *p* < 0.01, respectively, in the independent *t* tests for mean differences between two different sampling periods

### BMI and WC distributions

**Fig. 1:**
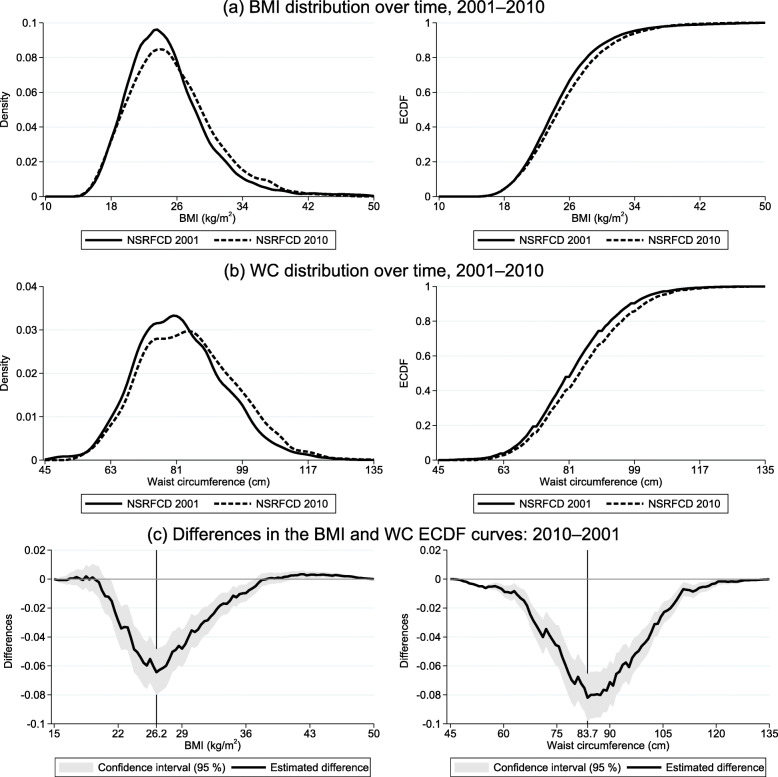
BMI and WC distribution over time. **a** BMI distribution: 2001–2010. **b** WC distribution: 2001-2010. **c** Differences in the BMI and WC ECDF curves: 2010–2001. BMI, body mass index; WC, waist circumstance

As illustrated by the BMI kernel density and empirical cumulative distribution function (ECDF) curve (Fig. 1), the BMI distribution disproportionately shifted rightward over the 2001–2010 period, with a rightward shift also discernible for WC. This rightward shift in both the BMI and WC distributions is confirmed by the Kolmogorov-Smirnov results (a combined K-S of 0.0683 and 0.0903, respectively, at *p* < 0.001). Graphing the 2001–2010 ECDF differences likewise reveals a clear rightward shift in the BMI distribution (Fig. 1), engendered mostly by negative differences. These latter, the largest of which include a BMI around 26 and a WC of approximately 84, reflect a reduction over the 9-year period in the proportion of individuals with normal body weight: around a 6% drop in the probability of a BMI < 26.

**Fig. 2:**
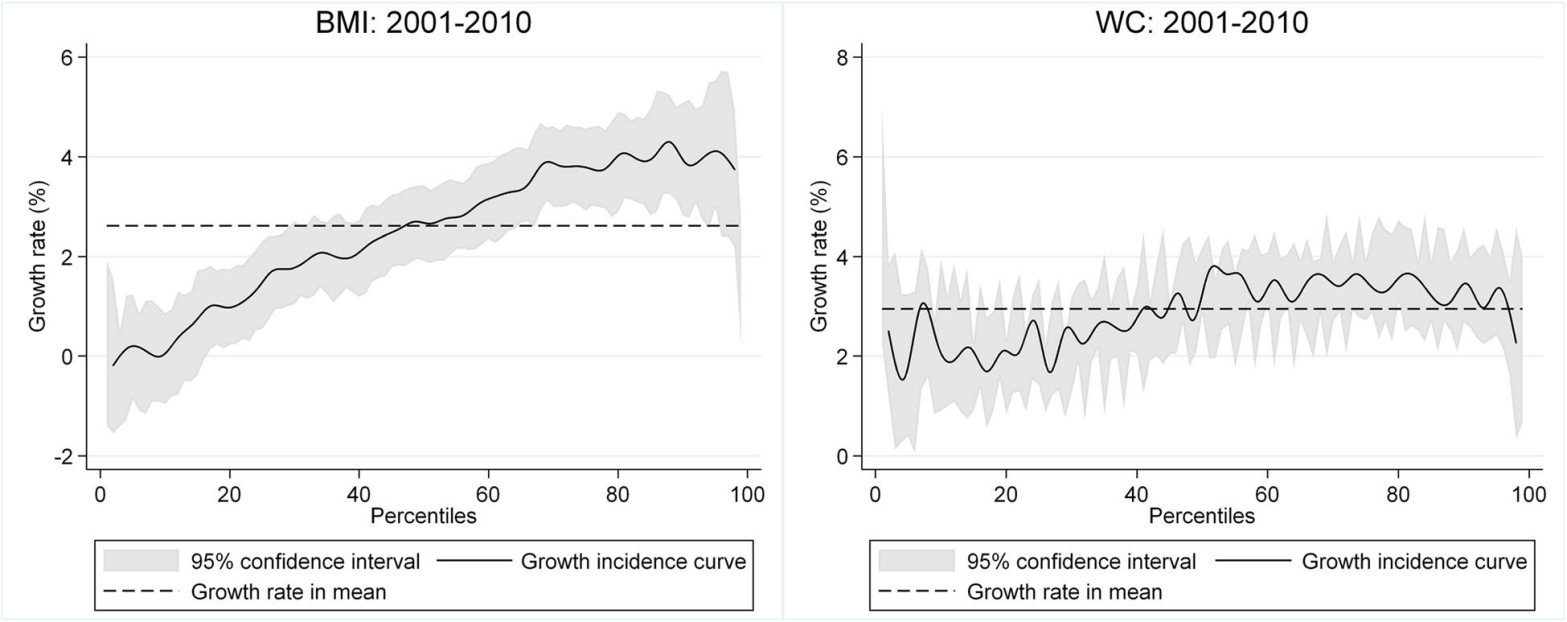
BMI and WC growth incidence curves. BMI, body mass index; WC, waist circumstance

To furnish additional insights into the magnitude of the body weight increase and which part of the weight distribution contributed more to overall growth, the growth incidence curves in Fig. 2 illustrate the percentage change at each percentile, with a horizontal line representing mean growth rate. The 2001–2010 time span is marked by a distributional skewing, with BMI growth higher at the upper end of the distribution. Only below the 40^th^ percentile are growth rates lower than the average, indicating a growing inequality in BMI over the period. The growth incidence curves for WC, in contrast, are quite flat.

### Decomposition of the total change in obesity prevalence

As Table 2 shows, general obesity rose by around 4 percentage points between 2001 and 2010, with 58% of the rise attributable to the growth component and about 42% to the redistributional effect. Hence, although the rising inequality outlined in Fig. 2 affected the rise in obesity rates, the general mean growth in BMI was more important. In contrast, the results of the Shapley decomposition, which takes into account the different central obesity thresholds for males and females, suggest that the increase in central obesity (8.6/6.2 percentage points for males/females) is due primarily to the mean growth component rather than the redistribution effect.

**Table 2:** Increase in general/central obesity prevalence decomposed into mean growth and redistribution components

Survey year	Difference	Growth component (G)	Redistribution component (R)	G/(G + R) (%)	R/(G + R) (%)
*General obesity*					
2001–2010	0.0373	0.0217 (0.0014)	0.0156 (0.0055)	58.1769	41.8231
*Central obesity*					
Male: 2001–2010	0.0864	0.0715 (0.0039)	0.0149 (0.0108)	82.7546	17.2454
Female: 2001–2010	0.0615	0.0700 (0.0043)	− 0.0085 (0.0107)	113.8211	− 13.8211

Standard errors are in parentheses

### Obesity inequality over time

Both the Gini and GE indices show a rise in BMI over the 2001–2010 period (Table 3), indicating that general obesity inequality worsened. However, whereas the Gini values indicate around a 4.6% increase, from 0.1046 for 2001 (95% CI 0.1032–0.1060) to 0.1094 for 2010 (95% CI 0.1071–0.1117); the GE index reflects only a moderate increase, with comparable magnitudes for GE(0) and GE(2). These observations imply that our finding of increasing obesity inequality remains robust irrespective of the relative importance attributed to the lower or upper tails of the distribution. The results for central obesity inequality over the study period are similar: the Gini index values increase from 0.0831 (95% CI 0.0821–0.0840) to 0.0852 (95% CI 0.0836–0.0868), albeit with a growth of only 2.5%. As Table 3 also shows, the magnitude of inequality in BMI is uniformly larger than that of WC.

**Table 3:** Intertemporal trends in obesity inequality

Survey year	Gini index	95% CI	Difference between t and t-1	% change between t-1 and t	Sensitive analysis	
					GE(0)	GE(2)
*BMI*						
2001	0.1046	0.1032–0.1060			0.0176	0.0190
2010	0.1094	0.1071–0.1117	0.0048^***^	4.5889	0.0188	0.0196
*WC*						
2001	0.0831	0.0821–0.0840			0.0109	0.0110
2010	0.0852	0.0836–0.0868	0.0021^***^	2.5271	0.0113	0.0114

CI denotes 95% confidence intervals; GE refers to generalized entropy. ^***^ indicates *p* < 0.01 in a *t* test for differences between the inequality indexes for two different sampling periods

According to Table 4, which shows the Gini coefficient for the two waves based on different demographics and socioeconomic status (SES), both males and females experienced a growth in general obesity inequality, with a rise also discernible among females aged 18–39. As for race, we found an increase in general obesity inequality among males across all racial groups as well as White females. This also applied to married or cohabiting partners for both sexes. Regarding education, a rise in general obesity inequality was observed in females with medium-level education, and males with medium- and high-level education. For central obesity inequality, sharp increases are observable among males, especially those aged 18–39, as well as among single females. For education, we also observed a growth in central obesity inequality among the medium-level educated.

**Table 4:** Trend in general obesity and central obesity inequality (Gini index) by gender, age, race, marital status, and education: 2001–2010

Subgroup			General obesity			Central obesity		
			2001	2010	Differences	2001	2010	Differences
Gender		Female	0.1112	0.1154	0.0042**	0.0853	0.0869	0.0017
		Male	0.0959	0.1017	0.0058***	0.0786	0.0810	0.0024*
Age	Female	18–39	0.1062	0.1119	0.0057*	0.0791	0.0821	0.0030
		40–59	0.1092	0.1118	0.0026	0.0805	0.0837	0.0032
		60+	0.1139	0.1140	0.0001	0.0851	0.0815	− 0.0036
	Male	18–39	0.0935	0.0978	0.0043	0.0752	0.0792	0.0040*
		40–59	0.0970	0.1013	0.0043	0.0783	0.0758	− 0.0025
		60+	0.0977	0.1031	0.0054	0.0779	0.0807	0.0028
Race	Female	White	0.1104	0.1152	0.0048**	0.0859	0.0872	0.0013
		Mulatto	0.1116	0.1164	0.0048	0.0837	0.0844	0.0008
		Black	0.1148	0.1132	− 0.0016	0.0842	0.0896	0.0054
	Male	White	0.0960	0.1006	0.0047**	0.0792	0.0809	0.0017
		Mulatto	0.0951	0.1029	0.0078*	0.0766	0.0806	0.0040
		Black	0.0953	0.1046	0.0093*	0.0737	0.0788	0.0051
Marital status	Female	Single	0.1056	0.1126	0.0070	0.0820	0.0877	0.0057*
		Married/living together	0.1098	0.1145	0.0047*	0.0846	0.0866	0.0020
		Widowed/separated/divorced	0.1131	0.1147	0.0016	0.0850	0.0822	− 0.0027
	Male	Single	0.0975	0.1014	0.0039	0.0733	0.0769	0.0035
		Married/living together	0.0932	0.0989	0.0057**	0.0774	0.0780	0.0006
		Widowed/separated/divorced	0.0960	0.0938	− 0.0021	0.0784	0.0742	− 0.0042
Education	Female	Low	0.1180	0.1185	0.0005	0.0882	0.0873	− 0.0009
		Middle	0.1091	0.1180	0.0089**	0.0844	0.0889	0.0045**
		High	0.1064	0.1107	0.0042	0.0801	0.0829	0.0027
	Male	Low	0.0997	0.1047	0.0050	0.0799	0.0789	− 0.0010
		Middle	0.0951	0.1006	0.0055*	0.0766	0.0817	0.0051***
		High	0.0930	0.1008	0.0078**	0.0800	0.0805	0.0005

^*^, ^**^ and ^***^ indicate *p* < 0.1, *p* < 0.05 and *p* < 0.01, respectively, in a *t* test for differences between the inequality measures at two different time points

### Decomposition of obesity inequality

Because the results given in Table 3 indicate no discrepancy between GE(0) and GE(2), a subsequent analysis adopted the Theil index (GE(1)) to decompose obesity inequality by gender, age, race, marital status, education, and combinations of these characteristics. Table 5 reports the results of this decomposition partitioned into within-group and between-group components. With all SES dimensions controlled for, general obesity inequality seems mostly attributable to within-group inequality (2001: 93.25%; 2010: 89.57%), with between-group inequality making only a small contribution (2001: 6.75%; 2010: 10.43%), accounted for mostly by age (2001: 1.87%; 2010: 3.50%) and marital status (2001: 1.97%; 2010: 2.89%). This pattern also holds for central obesity inequality, but with a higher contribution of between-group to total inequality (2001: 12.54%; 2010: 15.25%).

**Table 5:** Within-group and between-group obesity inequality

Survey year	GE(1)^a^		Gender	Age	Race	Marital status	Education	All SES
General obesity								
2001	0.0181	Within-group	0.9887	0.9813	0.9996	0.9803	0.9997	0.9325
		Between-group	0.0113	0.0187	0.0004	0.0197	0.0003	0.0675
2010	0.0190	Within-group	0.9940	0.9650	0.9992	0.9711	0.9997	0.8957
		Between-group	0.0060	0.0350	0.0008	0.0289	0.0003	0.1043
Central obesity								
2001	0.0109	Within-group	0.9774	0.9403	0.9981	0.9765	0.9959	0.8746
		Between-group	0.0226	0.0597	0.0019	0.0235	0.0041	0.1254
2010	0.0113	Within-group	0.9740	0.9368	0.9981	0.9664	0.9975	0.8475
		Between-group	0.0260	0.0632	0.0019	0.0336	0.0025	0.1525

Within- and between-group obesity inequality is reported as a proportion of overall inequality. Gender: male vs. female; age: 18–39, 40–59, 60 or higher; race: White, Mulatto, Black; marital status: single, married/living together, widowed/separated/divorced; education: low (illiterate/primary school), medium (secondary school/qualified worker/technical school), and high (university)

^a^GE(1) denotes the Theil index

## Discussion

Obesity follows a socioeconomic gradient [30] mainly because it entails the process of complex interactions between multiple environmental, economic, and social factors leading to physical inactivity and unhealthy diets [31]. Since obesity is related to a range of chronic diseases such as cardiovascular disease, stroke, type 2 diabetes, and a subset of cancers [32], SES differences in obesity will result in broader SES inequalities in health. These widening SES differentials in obesity thus imply that we should anticipate increasing inequalities in obesity-related diseases [33]. Consequently, obesity inequality serves as an important driver for the future development of inequalities in health and longevity [34]. Additionally, although obesity inequality generally serves as an important indicator of well-being—a multidimensional domain that mostly includes not only income but health, nutrition and education [11], it enables us to capture the allocation of resources across individuals and assess the effectiveness of policies combating obesity [35, 36]. It is also worthwhile to note that obesity negatively affects subjective well-being via deterioration in health, lower self-esteem or lower social acceptance [37]. Furthermore, it may also influence self-confidence, depression, personal and social relationships, and attitudes [37–39]. Thus, a rise in obesity inequality could also accentuate obesity-related stigma and discrimination [11]. Given the importance of obesity inequality, this present study is the first to examine changes of bodyweight distributions and obesity inequality in Cuban adult population.

Overall, our study identified the presence of a clear rightward shift in both the BMI and WC distributions, to which most of the increase in general (56%) and central obesity (82/114% for male/female) can be attributed. It also, however, identified a certain degree of distributional left-skewing reflecting about 4.6% and 2.5% of the general and central obesity inequality increases, respectively. The rise in BMI-based obesity inequality, which is particularly pronounced among males, Blacks, those aged 18–39, married or cohabiting partners, and individuals with medium- and high-level education, appears to have been driven by within-group rather than between-group inequality.

Although previous studies for US adults have also documented increases in both BMI and WC in the upper tails of their distributions [25, 40], the relatively small and even negative redistribution effect for central obesity in Cuban females suggests only a slow increase in (central) obesity inequality for females, with a WC that may have even stalled. For urban Cuba, the increases in both the Gini and GE coefficients indicate that obesity inequalities rose over the 2001-2010 period, although the 2010 Gini values of 0.109 and 0.085 for general and central obesity, respectively, are still lower than the US coefficient of 0.126 for general obesity in 2011–2014 [10]. Nonetheless, the magnitude of general obesity inequality in both urban Cuba and the US is much higher than that for China, whose Gini coefficients for 2011 range from 0.0823 to 0.0708 [11]. The analysis also provides evidence that, as in the US [10] and China [11], obesity inequality tended to be more pronounced among younger adults. In fact, according to the growth incidence curves for the different demographic and SES groups (see Additional files 2 and 3), these rapid increases in obesity inequality were driven primarily by distributional left-skewing. At the same time, the rise in aggregate inequality was mostly attributable to within-group rather than between-group inequality, suggesting that, as also found for the US [10] and China [11], the increase in overall obesity inequality was being driven not by changes in the demographic structure but rather by a population-wide increase across all subpopulations.

This study had some potential limitations. First, the study was admittedly limited by the relatively short (9-year) time period over which body weight distribution and obesity inequality in Cuba were tracked. Second, the available data were also about a decade old and thus did not capture recent economic changes in the country. Finally, a large share of missing values in the income data also prevented exploration of the heterogeneity in obesity inequality by different income level.

The variations in obesity inequality levels among urban Cuban adults have important implications for obesity outcomes, with rising inequality levels particularly affecting individual well-being at the right tail of the body weight distribution, where body weight tends to increase more quickly than the population average. These individuals are thus most likely to deviate from the socially perceived ideal, a deviation whose size may well determine obesity’s negative effects on well-being [41]. Hence, policy interventions to combat obesity during the early transition should primarily target groups experiencing the most rapid growth in inequality. Focusing on these groups is also important to avoid spillovers from strong peer effects at the upper end of the bodyweight distribution that could lead to rising obesity levels (i.e., a rightward distributional shift). Targeted policy interventions could thus profit from the so-called social multiplier effect [42, 43], that is, the externality inherent in peer effects. This effect implies that although obesity prevalence and inequality are likely to increase quickly in the early period of an obesity epidemic, targeted policy interventions can be relatively effective. As the epidemic spreads and obesity becomes a population-wide phenomenon, however (represented mainly by a rightward distributional shift), norms and ideals begin to change, making higher bodyweight levels more socially acceptable and even desirable. Then, not only do obesity’s stigmatizing effects seem less important, but the changing norms and ideals contribute strongly to obesity’s persistence, making policy interventions less effective.

## Conclusions

Overall, this study has shown that even though the objective health outcomes in Cuba are relatively good and not strongly dependent on SES characteristics [20], Cuba has been experiencing the same rising prevalence and inequality of obesity observed in other countries (e.g., the US and China). This observation implies that the obesity problem in Cuba will develop much as in the US, with obesity inequality rising on a par with the increase in obesity prevalence, then gradually leveling off, and eventually declining once a majority share of the population becomes obese. One aspect underscored by this study is that the observed rise in obesity inequality appears quite immune to Cuba’s highly egalitarian health care system, with its free universal access to high-quality primary care. Future research should thus pay attention not only to the consequences of increased obesity prevalence but also the ways in which the increase in obesity inequality affects individuals over the entire BMI distribution [10].

## Supplementary Information

**Additional file 1.** Shapley decomposition, obesity inequality measures, and GE-based decomposition

**Additional file 2: Figure A1.** BMI growth incidence curves by age, gender, race, marital status, and education

**Additional file 3: Figure A2.** WC growth incidence curves by age, gender, race, marital status, and education

## Data Availability

The datasets used and analyzed during the current study are available from the corresponding author on reasonable request.

## Abbreviations

BMI: Body mass index
CI: Confidence interval
ECDF: Empirical cumulative distribution function
GNI: Gross national income
GE: Generalized entropy
IDF: International Diabetes Federation
NSRFCD: National Survey on Risk Factors and Chronic Diseases
SES: Socioeconomic status
US: United States
WC: Waist circumference
WHO: World Health Organization
