# Supplementary material for: Inequality of weight status in urban Cuba: 2001–2010
Source: Popul Health Metr. 2021 May 4;19:24. doi: 10.1186/s12963-021-00251-6 (PMC8097838; doi:10.1186/s12963-021-00251-6)
Supplement: Supplementary file 1 — Additional file 1. Shapley decomposition, obesity inequality measures, and GE-based decomposition [file 12963_2021_251_MOESM1_ESM.docx]

**Additional file 1**

**Shapley decomposition**

The Shapley decomposition disentangled the total change in obesity prevalence into a mean-growth and a redistribution component in order to assess how much of the obesity increase is driven by a horizontal shift in bodyweight distribution (i.e., an increase in mean BMI or WC) and how much by a change in distribution pattern (e.g., an increased skewness towards the upper tail of the BMI or WC distribution) (Pak et al., 2016). Specifically, the obesity rate at time *t* can be expressed as follows:

$${OB}_{t}=OB(T|m_{t};c_{t}) (1)$$

where ${OB}_{t}$ represents obesity prevalence, and $T$ is the obesity threshold (30 for BMI-based obesity, 90 for male WC-based obesity, and 80 for female WC-based obesity). m is the average BMI and c is the Lorenz curve denoting the CDF of the BMI probability distribution.

Changes in obesity rates between $t_{n}$ and $t_{n-1}$ can be decomposed as:

$${OB}_{t_{n}}-{OB}_{t_{n-1}}=G\left( t_{n-1},t_{n} \right)+R\left( t_{n-1},t_{n} \right)+\varepsilon\left( t_{n-1},t_{n} \right) (2)$$

where $G(\cdot)$, $R(\cdot)$, and $\varepsilon(\cdot)$ represents the increase, redistribution, and residual parts, respectively. Specifically, the increase and redistribution parts are defined as follows:

$$G=OB\left( T | m_{t_{n}};c_{t_{n-1}} \right)- OB\left( T | m_{t_{n-1}};c_{t_{n-1}} \right) (3)$$

$$R=OB\left( T | m_{t_{n-1}};c_{t_{n}} \right)- OB\left( T | m_{t_{n-1}};c_{t_{n-1}} \right) (4)$$

where $G$ denotes the change in obesity prevalence attributable to a horizontal shift of the bodyweight distribution when keeping relative position constant. $R$ indicates the observed change in relative position while the average bodyweight remains constant. Although growth-inequality decomposition technique allows us to decompose total change into three major components, a better approach would decompose the change in obesity prevalence exactly into the first two terms only, that is

${OB}_{t_{n}}-{OB}_{t_{n-1}}=G^{s}\left( t_{n-1},t_{n} \right)+R^{s}\left( t_{n-1},t_{n} \right) (5)$

where $G^{s}$ and $R^{s}$ denote the Shapley values for the “increase” and “distribution” components of changes in obesity prevalence. These can be expressed as follows (Shorrocks, 2013):

$$G^{s}=\frac{1}{2}[OB\left( T | m_{t_{n}};c_{t_{n-1}} \right)- OB\left( T | m_{t_{n-1}};c_{t_{n-1}} \right)]+\frac{1}{2}[OB\left( T | m_{t_{n}};c_{t_{n}} \right)-OB\left( T | m_{t_{n-1}};c_{t_{n}} \right)] (6)$$

$$R^{s}=\frac{1}{2}[OB\left( T | m_{t_{n-1}};c_{t_{n}} \right)- OB\left( T | m_{t_{n-1}};c_{t_{n-1}} \right)]+\frac{1}{2}[OB\left( T | m_{t_{n}};c_{t_{n}} \right)- OB\left( T | m_{t_{n}};c_{t_{n-1}} \right)] (7)$$

In equations 6 and 7, the Shapley decomposition takes an equally weighted average of two decompositions, one at the reference year and the other at a later year (Shorrocks, 2013).

**Obesity inequality measures**

Gini and generalized entropy (GE) measures track the cardinal changes in obesity inequality. The Gini coefficient, a measure of statistical dispersion in a particular distribution, is a popular and widely used index measuring inequality (Yitzhaki, 1983). This measure is expressed as

$${Gini}_{t}=\frac{2}{m_{t}N_{t}^{2}}\sum_{i=1}^{N_{t}} {BMI}_{it}r_{it}-\frac{N_{t}+1}{N_{t}} (8)$$

where *N* is the sample size, *m* is the average BMI, ${BMI}_{it}$ is the individual BMI value at time *t*, and $r_{it}$ denotes the ranking of *i*^th^ BMI at time *t* in ascending order (with an equivalent expression for WC).

Because the Gini index is sensitive to changes around the distributional mode, we also adopted GE measures that are flexible enough to allow greater sensitivity away from the distributional middle (Shorrocks, 1984; Yang, 1999). We express the GE index as

$${GE}_{t}\left( \omega\right)\frac{1}{w\left( w-1 \right)}[\frac{1}{N_{t}}\sum_{i=1}^{N_{t}} ({\frac{{BMI}_{it}}{m_{t}})}^{w}-1] (9)$$

where $\omega$ is a scaling parameter representing the weight given to distances between individual BMI at different parts of the BMI distribution (with the same equation used for WC). The mean logarithmic deviation (MLD) is the limiting case when $\omega=0$ (GE(0)), while the Theil index is the limiting case when $\omega=1$ (GE(1)) (Cowell and Flachaire, 2015), which assures equal treatment of the differences between individual BMI levels at different parts of the BMI distribution. GE(2) is half the square of the coefficient of variation (Jenkins and Kerm, 1999).

**GE-based decomposition**

The GE-based decomposition by subgroup splits the GE index into within-group and between-group inequality (Shorrocks, 1984):

$${GE}_{t}\left( \omega\right)={{GE}_{t}\left( \omega\right)}_{within-group}+{{GE}_{t}\left( \omega\right)}_{between-group}, {{GE}_{t}\left( \omega\right)}_{within-group}=\sum_{j} \frac{{BMI}_{t,j}}{{BMI}_{t}}{GE}_{t,j},$$

${{GE}_{t}\left( \omega\right)}_{between-group}=\sum_{j} \frac{{BMI}_{t,j}}{{BMI}_{t}}ln(\frac{{{BMI}_{t,j}}/{{BMI}_{t}}}{{N_{t,j}}/{N_{t}}}) (10)$

where ${GE}_{t,j}$ and ${BMI}_{t,j}$ represent the GE index and BMI at subgroup j and time t, respectively (with a similar approach applied for WC). In equation 10, ${{GE}_{t}\left( \omega\right)}_{within-group}$ denotes the weighted sum of inequality within groups, while ${{GE}_{t}\left( \omega\right)}_{between-group}$ designates the component driven by the heterogeneity in inequality between groups.

Cowell FA, Flachaire E. Chapter 6 - Statistical Methods for Distributional Analysis. In: ATKINSON, A. B. & BOURGUIGNON, F. (eds.) Handbook of Income Distribution. North Holland: Elsevier, 2015.

Jenkins SP, Kerm PV. sg107: Generalized Lorenz curves and related graphs. *Stata Technical Bulletin* 1999; **48**: 25–9.

Pak T-Y, Ferreira S, Colson G. Measuring and tracking obesity inequality in the United States: Evidence from NHANES, 1971-2014. *Population Health Metrics* 2016; **14**: 12.

Shorrocks AF. Decomposition procedures for distributional analysis: A unified framework based on the Shapley value. *The Journal of Economic Inequality* 2013; **11**: 99-126.

Shorrocks AF. Inequality Decomposition by Population Subgroups. *Econometrica* 1984; **52**: 1369-85.

Yitzhaki S. On an extension of the Gini inequality index. *International Economic Review* 1983; **24**, 617-628.

Yang DT. Urban-biased policies and rising income inequality in China. *American Economic Review* 1999; **89**: 306-310.
