# Supplementary material for: Inequality of weight status in urban Cuba: 2001–2010
Source: Popul Health Metr. 2021 May 4;19:24. doi: 10.1186/s12963-021-00251-6 (PMC8097838; doi:10.1186/s12963-021-00251-6)
Supplement: Supplementary file 3 — Additional file 3: Figure A2. WC growth incidence curves by age, gender, race, marital status, and education [file 12963_2021_251_MOESM3_ESM.docx]

**Additional file 3**


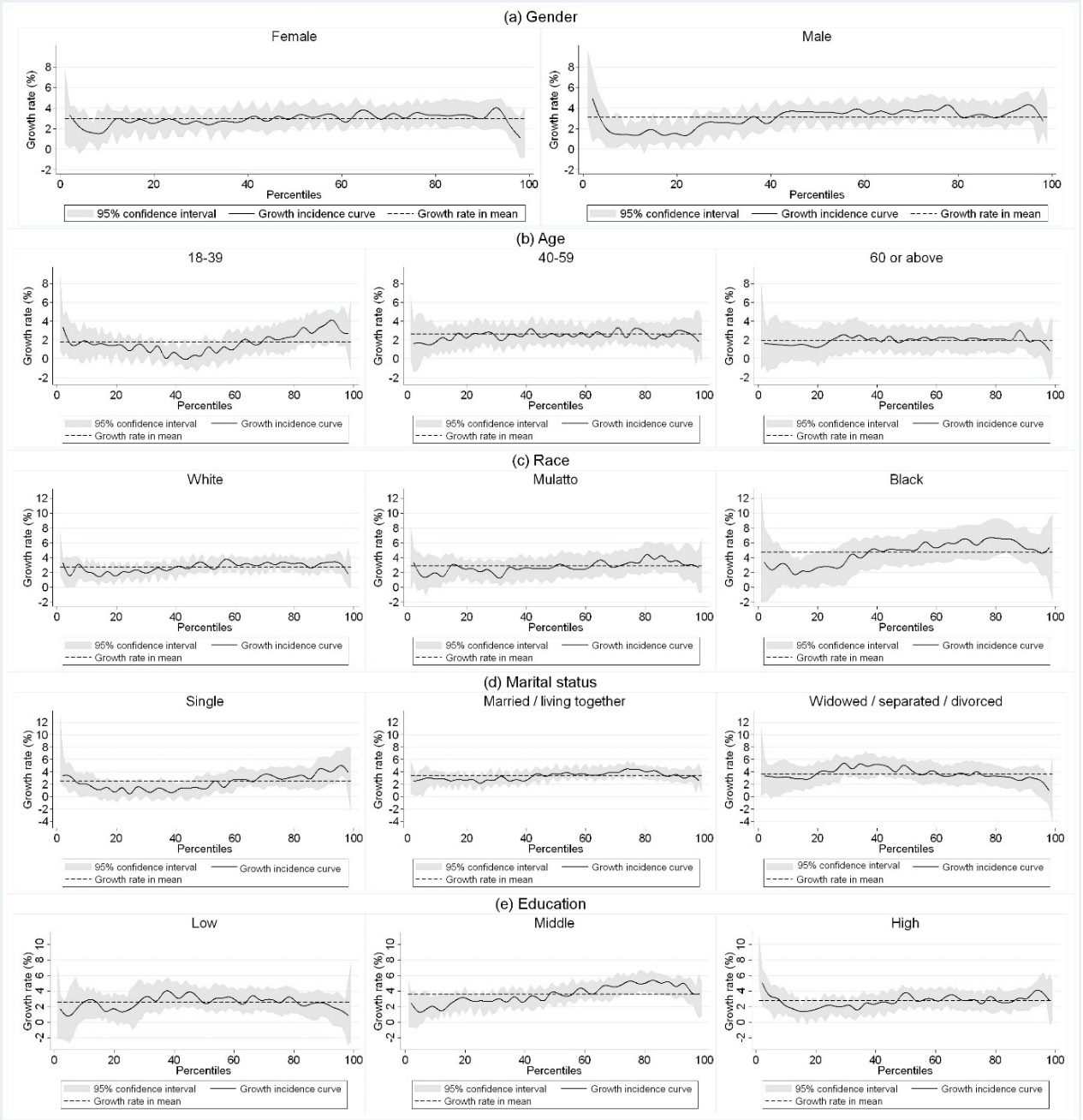


**Figure A2.** WC growth incidence curves by age, gender, race, marital status, and education.
